# Genome assembly of the southern pine beetle (*Dendroctonus frontalis* Zimmerman) reveals the origins of gene content reduction in *Dendroctonus*

**DOI:** 10.1098/rsos.240755

**Published:** 2024-12-11

**Authors:** Megan Copeland, Shelby Landa, Adekola Oluwatosin Owoyemi, Michelle M. Jonika, James M. Alfieri, J. Spencer Johnston, Terrence Pradakshana Sylvester, Bethany R. Kyre, Zachary Hoover, Carl E. Hjelmen, Lynne K. Rieske, Heath Blackmon, Claudio Casola

**Affiliations:** ^1^ Department of Biology, Texas A&M University, College Station, TX, USA; ^2^ Department of Ecology and Conservation Biology, Texas A&M University, College Station, TX, USA; ^3^ Department of Molecular Biosciences, The University of Texas at Austin, Austin, TX, USA; ^4^ Department of Entomology, Texas A&M University, College Station, TX, USA; ^5^ Department of Biological Sciences, The University of Memphis, Memphis, TN, USA; ^6^ USDA Forest Service, Forest Health Protection, San Bernardino, CA, USA; ^7^ Department of Biochemistry, Texas A&M University, College Station, TX, USA; ^8^ Department of Biology, Utah Valley University, Orem, UT, USA; ^9^ Department of Entomology, University of Kentucky, Lexington, KY, USA; ^10^ Interdisciplinary Doctoral Program in Ecology and Evolutionary Biology, Texas A&M University, College Station, TX, USA; ^11^ Interdisciplinary Graduate Program in Genetics & Genomics, Texas A&M University, College Station, TX, USA

**Keywords:** bark beetles, Stevens elements, gene family, gene loss, gene annotation, transposable elements

## Abstract

*Dendroctonus frontalis* also known as southern pine beetle (SPB), is the most damaging insect forest pest in the southeastern United States. Genomic data are important to provide information on pest biology and to identify molecular targets to develop improved pest management approaches. Here, we produced a chromosome-level genome assembly of SPB using long-read sequencing data. Synteny analyses confirmed the conservation of the core Coleopteran Stevens elements and validated the *bona fide* SPB X chromosome. Transcriptomic data were used to obtain 39 588 transcripts corresponding to 13 354 putative protein-coding loci. Comparative analyses of gene content across 14 beetles and three other insects revealed several losses of conserved genes in the *Dendroctonus* clade and gene gains in SPB and *Dendroctonus* that were enriched for loci encoding membrane proteins and extracellular matrix proteins. While lineage-specific gene losses contributed to the gene content reduction observed in *Dendroctonus*, we also showed that widespread misannotation of transposable elements represents an important cause of the apparent gene expansion in several non-*Dendroctonus* species. Our findings uncovered distinctive features of the SPB gene complement and disentangled the role of biological and annotation-related factors contributing to gene content variation across beetles.

## 1. Background

Bark beetles (Scolytinae: Curculionidae: Coleoptera) are common forest pests responsible for the annual loss of millions of conifers and other trees worldwide [1–3]. The genus *Dendroctonus* (Latin for ‘tree killer’) includes several bark beetle species that can spawn large outbreaks and are capable of colonizing and overwhelming both weakened and healthy trees [4]. Although bark beetle population bursts represent natural forest disturbance events [5], the ecological repercussions of large-scale *Dendroctonus* outbreaks can be severe, including ecosystem degradation, hydrological instability, reduced carbon sequestration and loss of revenue associated with commercial and recreational use [6,7].

The southern pine beetle (SPB) *Dendroctonus frontalis* Zimmerman has historically been associated with the most severe bark beetle epidemics in the southeastern United States, leading to the loss of millions of hectares of managed and unmanaged conifer forests [8,9]. SPB infestations are initiated by female pioneer beetles that bore into the phloem of host trees recognized using visual and chemical cues [8,10]. Adult female beetles lay their eggs in the burrows within the phloem, where larvae will hatch and feed on the surrounding vascular tissue, expanding the burrows into larger galleries [4]. Females may also emerge from the host before laying all their eggs, seek another host, and lay another brood. Upon reaching maturity, the adult beetles find a new host tree and continue to propagate [4,11].

Under ideal conditions, a population could produce up to eight generations within one year, leading to a potential for rapid population increase in an impacted area [12]. When female pioneer beetles find a new host tree, they release frontalin, an aggregation pheromone that, along with the host tree’s distress odours, attracts more SPB males and females. A process critical for overcoming the host tree’s defences as the number of attacking beetles is positively correlated with the host tree’s strength [13]. Changing climate patterns, including warming temperatures and fluctuating precipitation patterns, together with a lack of effective management strategies, have allowed an unprecedented northward range expansion of SPB [14].

Traditional-integrated management strategies for bark beetle pests, including population density surveys, outbreak prevention and treatment of affected areas are costly and pose logistic challenges over large areas [12,15,16]. Given the geographical expansion of SPB and other *Dendroctonus* species coupled with their persistent outbreaks over historic geographic ranges, additional tools are needed to develop innovative strategies for the management of these bark beetles. Genomic data are increasingly recognized as critical resources to study pesticide resistance and susceptibility mechanisms [17] and facilitate identifying the genetic basis of species-specific adaptations, including the suite of phenotypes associated with the tree-killing habit of SPB and several other *Dendroctonus* species.

Furthermore, genomic resources are essential to understanding the evolution of chromosome number and gene content variation, two fundamental sources of genetic variation that underlie adaptive evolution. The genus *Dendroctonus* shows a particularly fast-evolving karyotype, with 2*n* = 30 being the presumed ancestral chromosome number that is still retained in a few species, and 2*n* = 12 being the smallest karyotype [4]. Several species experienced lineage-specific fusions of autosomes and ancestral sex chromosomes, leading to the formation of neo-XY chromosomes [18]. The extreme karyotypic variation in *Dendroctonus* is further supported by the presence of chromosome number changes between populations of the same species. For instance, the two *D. frontalis* morphotypes A and B exhibit the karyotypes 7AA + XY and 5AA + XY, respectively [19].

Conversely, the genome sequencing of two *Dendroctonus* species, *D. ponderosae* Hopkins (mountain pine beetle or MPB) and *Dendroctonus valens* LeConte (red turpentine beetle or RTB), have shown a stable gene number of approximately 13 000 [20–22]. This is surprisingly lower than what is reported in most other beetles and, more broadly, insects. Intriguingly, two other sequenced bark beetle species, *Ips typographus* Linnaeus (European spruce bark beetle) [23], and *Hypothenemus hampei* Ferrari (coffee berry borer) [24,25] contained between 19 000 and 23 000 genes. The more distantly related wood-boring species *Anoplophora glabripennis* Motschulsky (Asian longhorned beetle) and *Agrilus planipennis* Fairmaire (emerald ash borer) shared a similarly high gene content [26]. The gene annotation of additional *Dendroctonus* genomes is key to verify this finding and determine the causes of gene content reduction in this genus.

With the goals of identifying genes underlying the biology of tree-killing bark beetles and investigating the peculiar chromosomal and gene content features of *Dendroctonus*, we generated a high-quality genome assembly and gene annotation resources for *D. frontalis*, using a combination of long-read sequencing, Omni-C scaffolding and high-throughput transcriptomic data. We identified synteny conservation, which refers to the preservation of large collinear blocks of sequences across genomes, between SPB’s largest scaffolds and chromosomes from other species. This includes the conservation of the putative SPB X chromosome with the MPB neoX chromosome. The comparative analysis of SPB and other beetle genomes revealed SPB- and *Dendroctonus*-specific gene gains and losses potentially associated with adaptations and an inflated gene count in several non-*Dendroctonus* beetles due to the erroneous annotation of transposable elements.

## 2. Material and methods

### 2.1. Biological material and nucleic acid extraction

SPB specimens were collected from infested loblolly pine trees in the Homochitto National Forest, MS (31°21'16.152" N, 90°49'42.678" W), between 29 September and 7 October 2019 (electronic supplementary material, table S1). Four females and three males were collected and stored frozen until DNA extraction was performed. High molecular weight (HMW) DNA was extracted from three female and two male pooled sample sets using the MagAttract High Molecular Weight kit (Qiagen, Valencia, CA) according to the manufacturer’s protocols with the addition of an extra wash step using the provided wash buffer. HMW Genomic DNA was collected from two additional pooled sample sets, one female and one male, using the Nanobind Tissue Big DNA Kit (Circulomics, Baltimore, USA) according to the manufacturer’s protocols.

RNA was obtained from three adult females, four adult males and 39 instars at various developmental stages stored in either RNAlater or liquid nitrogen after collection and subsequently maintained at −80°C until shipment for sequencing (electronic supplementary material, table S1). Total RNA was isolated from whole beetles with TRI Reagent RT (Molecular Research Center Inc., Cincinnati, OH). RNA integrity was verified using gel electrophoresis and absorbance was measured at 260/280 and 230/280. cDNA was synthesized using SuperScript™ III Reverse Transcriptase (Invitrogen, Carlsbad, CA) according to the manufacturer’s instructions at a concentration of 3000 ng ml^−1^ and used as a template for the RT-qPCR standard curve, constructed using a fivefold dilution.

### 2.2. Genome sequencing

Sequencing was carried out at the Texas A&M Institute for Genome Sciences and Society Core facility. The Oxford Nanopore sequencing platform was used to generate long-read sequencing. Long-read sequences were obtained utilizing the SQK-LSK109 reaction kit, and libraries were prepared following the manufacturer’s protocol. One R9.4.1 flow cell was used for each specimen, and base-calling was performed with Guppy (v. 3.2.10) using default system settings. Sequencing yielded a total of 39 GB of read data (approx. 198× coverage).

### 2.3. Omni-C sequencing

Dovetail Genomics prepared one Omni-C library and performed sequencing (Dovetail Genomics, CA). For each Dovetail Omni-C library, chromatin was fixed in place with formaldehyde in the nucleus. Fixed chromatin was digested with DNaseI and then extracted. Chromatin ends were repaired and ligated to a biotinylated bridge adapter, followed by proximity ligation of adapter-containing ends. After proximity ligation, crosslinks were reversed, and the DNA was purified. Purified DNA was treated to remove biotin that was not internal to ligated fragments. Sequencing libraries were generated using NEBNext Ultra enzymes and Illumina-compatible adapters. Biotin-containing fragments were isolated using streptavidin beads before PCR enrichment of each library. The library was sequenced on an Illumina HiSeqX platform (150 bp paired-end reads) to produce approximately 30× sequence coverage.

### 2.4. Genome size estimation

Flow cytometric methods following [27] were used to determine the *D. frontalis* genome size. Neural tissue from individual frozen samples of *D. frontalis* was dissected and deposited into 1 ml of Galbraith buffer. All samples were co-prepared with a standard (lab stock of *Drosophila virilis*, genome size = 328 Mbp). Samples were gently ground with a Kontes ‘A’ pestle approx. 15 times to release nuclei. After passing samples through 41 μm mesh filters, samples were stained with 25 µl of 1 mg µl^−1^ propidium iodide and incubated in a dark refrigerator. Samples were run on a Beckman Coulter CytoFlex flow cytometer with a 488 nm blue laser. Means of 2C nuclei fluorescence peaks were measured for both sample and standard using gating methods supplied within the instrument’s software before calculating the estimated genome size.

### 2.5. Genome assembly

We assembled the female SPB genome using all female reads (approx. 19 Gb of reads) in Flye version 2.8.2-b1689 with default settings [28]. We then used Blobtools version 1.1.1 to remove potential contaminants [29]. Blobtools require three inputs—assembly, coverage and hits. First, we mapped the raw reads back to the assembled genome using minimap2 version 2.20-r1061 with default settings to generate the coverage input [30]. We then used the blastn module from NCBI BLAST+ version 2.12.0 to find sequence similarities between the assembled genome and 39 eukaryote and bacteria genomes (retrieved 25 October 2021; electronic supplementary material, table S2), which generated the hits input [31]. Finally, we combined the assembly, coverage, and hits inputs using Blobtools version 1.1.1 to visualize and remove contaminant sequences. Contaminant sequences were classified as those sequences with abnormal coverage and GC proportions compared with the rest of the genome and having higher similarity with prokaryotic sequences.

### 2.6. Omni-C scaffolding

InstaGRAAL was used for scaffolding the Dovetail Omni-C reads to the long-read contigs produced by the Flye assembler [32]. The data were prepared with hicstuff v. 3.1.0, using BWA as the aligner, and the enzyme option was set to ‘mnase’ to be compatible with the Omni-C data. Additionally, the filter setting was turned on to filter any short-range mapping events [33]. Since no Omni-C-induced errors were detected, scaffolds were not improved by polishing.

### 2.7. Characterization of repetitive sequences

Simple sequence repeats (SSRs) were identified with the R package micRocounter [34]. The minimum number of repeated motifs to be considered an SSR was six for dinucleotides; four for trinucleotides and three for tetra-, penta- and hexanucleotides. A maximum gap to continue an SSR array was set to one nucleotide. Larger tandem repeats were identified using TRF v. 4.09.1 [35]. The parameters were set as follows: a matching weight of two, a mismatch penalty of five, an indel penalty of seven, a match probability of 80, an indel probability of 10, a minimum alignment score of 50 for reporting, and a maximum period size for reporting of 2000. The maximum expected length of any repeat array was set to 10 Mbp. EDTA v. 2.0.0 [36] was used with the ‘sensitive’ parameter set to 1 to construct a library and annotate interspersed repeats across the genome assembly. Identity with the consensus sequence of transposable elements identified by homology was an output of EDTA.

R scripts incorporating ape [37] and SeqinR [38] were used to compile and split overlapping repeat annotations. Partially overlapping repeats were split 50/50. Fully overlapping repeat annotations were split 25/50/25, with the first and last 25% of the overlapping region attributed to the larger repeat. Plots were made using ggplot2 [39].

### 2.8. Read coverage and synteny analysis

We calculated the normalized read coverage of each scaffold in RStudio [40] and used the average genomic coverage to identify the scaffold that probably represents the X chromosome. To assess assembly quality, syntenic regions between the *D. frontalis* assembly and the new female assembly of *D. ponderosae* [21] were visualized with Circos v. 0.69-9 [41]. Repetitive sequences were masked by running EDTA v. 2.0.0 [36] on the genome assembly. Scaffolds and contigs under 2 Mbp were removed using SeqKit [42] before creating the necessary karyotype files. Genome alignments were obtained using minimap2 v. 2.24 [30], and the resulting output file was then used to create a links file. This links file was used to generate the Circos plot. Conservation of Stevens elements was visualized using the same genome alignment data and the RIdeogram package [43].

### 2.9. Transcriptome sequencing and assembly

cDNAs from SPB specimens were sequenced on an Illumina MiSeq instrument using both 2 × 75 bp and 2 × 150 bp reads (electronic supplementary material, table S3). Quality assessment of the data was performed using FastQC [44]. TrimGalore [45] was used to remove reads from the dataset that had a Phred quality score below 30 and were shorter than 20 bp. After low-quality reads were removed, contaminant sequences were identified using FastqScreen. The small size of the organism necessitated extracting RNA from whole-body samples, and contaminant sequences from the gut microbiome or SPB symbionts may have been present. After contaminants were identified, the RNAseq reads were mapped to contaminant genomes with the Burrows–Wheeler alignment tool [46] and filtered according to map quality. rRNA contamination was also removed by mapping the RNAseq reads to a comprehensive set of Coleopteran rRNA sequences retrieved from the SILVA rRNA gene database [47]. The remaining reads should represent only mRNA expressed by female, male and larval SPB samples.

Transcriptome assembly was carried out using the Trinity *de novo* assembly pipeline [48]. To remove redundancy, transcripts were subsequently clustered using the cd-hit-est tool available through the CD-HIT software package [49]. The TransDecoder [50] pipeline, which leverages BLAST [31] and Pfam [51] evidence, was used to identify transcripts in both the full and reduced assemblies that represent the longest open reading frame (ORF). TransDecoder filters out smaller isoforms and spurious or chimeric assemblies. The final draft assembly is a complete, non-redundant set of transcripts expressed by *D. frontalis*.

### 2.10. Removal of transcripts containing transposable elements

To remove putative transcripts encoded by transposable elements (TEs), we performed a BLAST search of transcript sequences against the SPB library of TEs using the following modified parameters: -ungapped-max_hsps 5-max_target_seqs 10-evalue 0.001. The BLAST results were merged using the merge program in the bedtools suite [52], retrieving 1893 transcripts with TE content. The 1070 transcripts with TE sequence coverage greater than or equal to 50% were removed.

### 2.11. Gene annotation

We used SPALN2 [53] to align the 40 493 transcripts on to the SPB genome assembly and mapped 39 588 transcripts, with the following parameters: -Q7-O6-t48-d. To determine the number of loci, we applied the program cluster in the bedtools suite [52] to exon and gene coordinates in the gff3 file, then identified for each cluster the main transcript by prioritizing ORF completeness (presence of both start and stop codons) and ORF length. Functional annotation of the 39 588 transcripts and the 13 354 loci was carried out using eggNOG-mapper v. 2 with default parameters [54].

### 2.12. Gene family analysis

Genome assembly, protein FASTA files and gff files of 17 gene sets were obtained from the NCBI genome database (electronic supplementary material, table S4). The gff files were used to identify the longest coding sequence/protein per locus and the corresponding transcript IDs, in order to avoid including multiple isoforms/proteins in loci with alternative transcript data in gene family size analyses. Protein sequence files were filtered according to this criterion, thus retaining only the longest protein for each gene. These sequence files were used to infer gene families using OrthoFinder with default settings [55]. Protein sequences of *Drosophila melanogaster* and *D. ponderosae* (MPB) genes belonging to orthogroups of interest were used for functional enrichment analyses in STRING [56].

We inferred gene family expansions and contractions along the phylogeny of the 14 beetles and three outgroup species using CAFE 4 [57]. Gene families with no variation across species, highly variable gene families (s.d. > 3) and families present in fewer than six species were removed, leaving a total of 8903 orthogroups analysed with CAFE. We ran the program with default parameters and one *λ*, as we did not have specific hypotheses to test regarding variation in the rate of gene gain and loss along the species phylogeny. Sequence similarity searches to verify gene losses were performed using the standalone version ncbi-blast−2.11.0+ of BLAST+ [31] with default parameters except -max_hsps 10-max_target_seqs 20-ungapped-comp_based_stats F-evalue 0.1.

### 2.13. Plant cell wall-degrading enzyme genes

Genes encoding for plant cell wall-degrading enzymes (PCWDEs) were identified in beetles by searching for the keywords ‘Pectinesterase’ and ‘Glyco_hydro’ for carbohydrate esterases (CE) and glycoside hydrolases (GH), respectively, in the eggNOG-mapper annotation. Polysaccharide lyase (PL) genes were retrieved by searching for ‘PL4’ in the CAZy database resuling in the eggNOG-mapper annotation. These genes were mapped on to the orthogroups from OrthoFinder. All genes from those orthogroups were then retrieved from the 17 analysed species.

### 2.14. Identification of transposable elements in gene sets

Protein domain names were retrieved using the PFMA results from the eggNOG-mapper v. 2 analysis described above. We screened domain names using the following TE-associated domain keywords: DDE, hAT, integrase, RVT, MULE, Retrotrans, rve, gag, Tnp, Helitron and THAP. Protein sequences containing these domains were retrieved and used for local searches against the corresponding genomes using the ncbi-blast−2.11.0+ version of BLAST+. The BLAST parameters were set to default except for -max_hsps 10-max_target_seqs 20-ungapped-comp_based_stats F-evalue 0.1. As control, gene representatives from the 12 non-TE gene families with the highest average gene count across all species were also analysed. Copy numbers for each gene were estimated by counting BLAST hits of at least 50 amino acids in non-overlapping genomic regions with the four possible combinations of distance between hits 20 or 50 kb and percentage identity 50% or 75%.

## 3. Results and discussion

### 3.1. Genome assembly

A high-quality SPB genome assembly was generated using approximately 19 Gbp of long-reads from female specimens, corresponding to a nearly 100× coverage of the 194.7 Mbp of the female *D. frontalis* estimated using flow cytometry. After removing potentially contaminating DNA sequences from symbiotic and commensal species, we obtained a *D. frontalis* genome assembly formed by 381 scaffolds at a total length of 173.7 Mbp, with a scaffold N50 of 24.8 MB. A total of 97.72% of the assembled genome localized in eight chromosome-level scaffolds between 12.4 and 42.5 Mbp. The discrepancy in the genome assembly and the genome size estimate is most likely the result of the software’s challenges in assembling the abundant, highly repetitive microsatellite sequences that are commonly found in Coleopterans’ genomes [58,59]. A total of approximately 4.4 Mbp were contained in the remaining 373 scaffolds with a length range of 1–124 Mbp. Compared with the reference genome assemblies for *D. ponderosae* and *D. valens*, *D. frontalis* exhibits a smaller genome size but a higher scaffold N50 (table 1). Gene set completeness analyses using BUSCO showed that 94.2% of the 2124 Endopterygota conserved orthologues are present in the SPB scaffolded assembly as complete copies.

**Table 1.** Assembly statistics for *D. frontalis* and the reference genome assemblies for *D. ponderosae* [21] and *D. Valens* [22].

assembly feature	*D. frontalis*	*D. ponderosae*	*D. valens*
genome assembly (Mbp)	173.7	223.6	322.4
GC content (%)	36.5	36.0	37.0
scaffold number	381	2112	922
scaffold N50 (Mbp)	24.8	16.6	1.7
scaffold L50	3	4	57

### 3.2. Repetitive sequence identification

**Figure 1.**
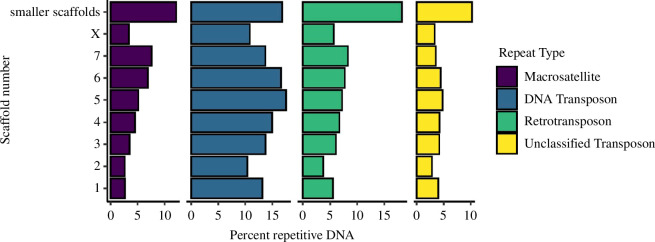
Distribution of repetitive elements. The vertical axis represents scaffold numbers, while the horizontal axis displays the percentage of each repetitive element type which is denoted by colour. Microsatellites were excluded from the plot as all values were less than 0.5%.

Twenty-eight per cent of the SPB-assembled genome was identified as repetitive. Chromosome-level scaffolds contained a lower proportion of repeats compared with small scaffolds, as expected given the challenges posed by repetitive DNA to the assembly of long pseudomolecules (figure 1). TEs formed approximately 23% of the assembled genome, similar to what was found in MPB [20,60] but lower than in the larger genome of *D. valens* [22]. Approximately 13% and approximately 6% of the SPB genome were formed by DNA transposons and retrotransposons, respectively (electronic supplementary material, table S5). We also identified 71 000 tandem repeat arrays contributing approximately 5% of the assembled genome. The telomeric (TTAGG)n repeat found in some members of Scolytinae [61] was not found at the termini of large scaffolds of the assembled genome.

### 3.3. Synteny conservation with mountain pine beetle and identification of the putative chromosome X in southern pine beetle

**Figure 2.**
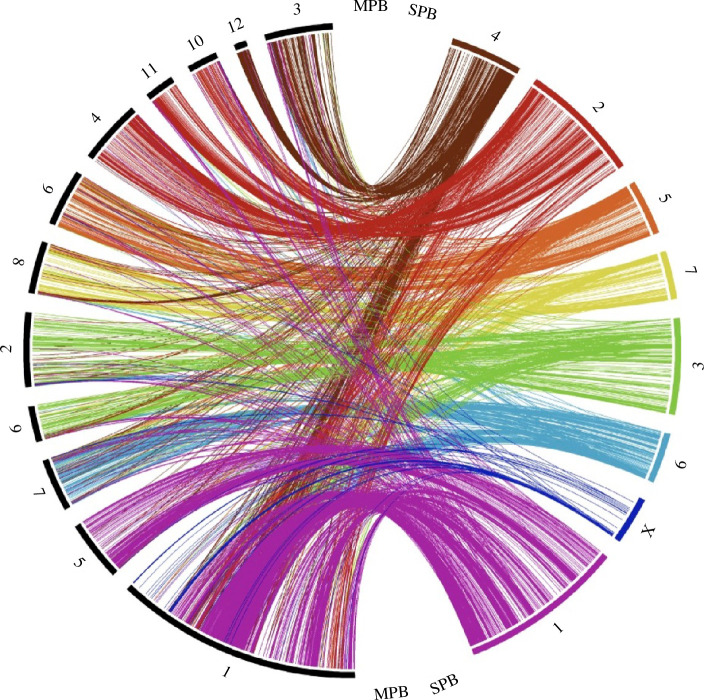
Synteny correspondence between *D. frontalis* and *D. ponderosae* chromosomes. The putative *D. frontalis* X chromosome shows synteny conservation with part of the neoX chromosome in *D. ponderosae*. *Dendroctonus frontalis* chromosomes: coloured segments. *Dendroctonus ponderosae* chromosomes: black segments.

After normalizing read coverage across the genome assembly of *D. frontalis* for female and male samples, we found a reduction in male read coverage in scaffold 8, suggesting that this scaffold represents the X chromosome (electronic supplementary material, figure S2). The comparison between the SPB and *D. ponderosae* genomes revealed a high level of synteny conservation. The scaffold containing the X chromosome in SPB maps to scaffold 1 in *D. ponderosae*, which corresponds to the neoXY system in *D. ponderosae* (figure 2).

**Figure 3.**
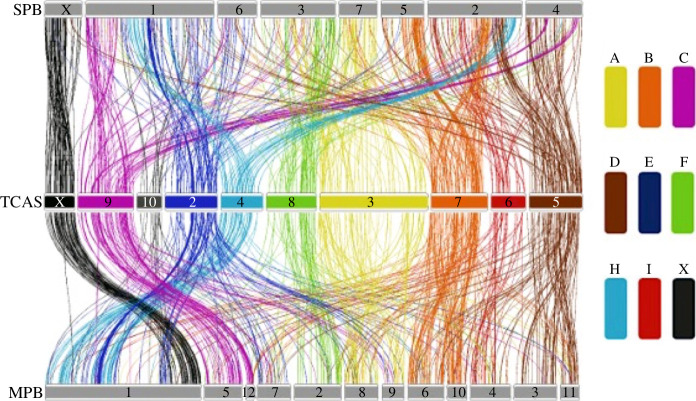
Synteny plot displaying conservation of Stevens elements. Each colour represents one of the nine Stevens elements identified by Bracewell *et al*. [62] and is labelled in the legend to the right. Each link represents a syntenic region between the genomes ordered as follows: top: *D. frontalis* (SPB), middle: *T. castaneum* (TCAS), bottom: *D. ponderosae* (MPB).

Based on the synteny analysis of *Tribolium castaneum* and five other beetles, Bracewell *et al*. [62] identified nine ancestral linkage groups, known as Stevens elements, that share a conserved set of genes across Coleoptera. A synteny plot based on *D. frontalis*, *D. ponderosae* and *T. castaneum* genomes shows the conservation of the nine Stevens elements (figure 3).

### 3.4. Gene annotation

RNAseq data from female and male adult beetles and instars were assembled and processed to generate a final transcriptome of 40 493 transcripts (see table 2 and §2). A total of 39 588 transcripts were mapped into the SPB genome assembly using SPALN [53], allowing to identify 13 354 non-redundant putative gene loci (table 2). Nearly 99% of SPB genes were annotated on the eight chromosome-level scaffolds. On average, SPB loci are 6683 bp long and contain 5.9 exons. A similar number of loci was identified in the recently improved assembly of *D. ponderosae* [21] and in the *D. valens* genome [22], whereas the genome of the other Scolytinae beetles *H. hampei* and *I. typographus* contains a significantly higher number of genes [23–25].

**Table 2.** SPB genome and transcriptome metrics and gene annotation completeness as indicated by the BUSCO pipeline proportion of single-copy and duplicated orthologues.

	genome assembly	TEs removed	SPALN mapped	loci
longest ORF transcripts	—	40 493	39 588	13 354
BUSCO % complete	94.2	96.0	94.9	92.2
BUSCO % fragmented	2.4	1.2	1.5	2.9
BUSCO % total	96.6	97.2	96.4	95.1

Among all mapped transcripts, 9678 (72.5%) were functionally annotated with eggNOG-mapper v. 2 [54] (electronic supplementary material, table S6). BUSCO analyses showed a slight decrease in SPB orthologues (complete and fragmented) from 97.2% to 95.1% between the transcriptome and the predicted loci (table 2). Given the predicted 13 354 loci, the maximum number of SPB genes according to the transcriptome BUSCO coverage of 97.2% is 13 649.

### 3.5. Gene family analyses

We investigated changes in the gene content that might be associated with trait evolution in SPB and *Dendroctonus*. Leveraging on high-quality genomic resources available for two non-SPB *Dendroctonus* species, two additional Scolytinae, nine other beetles and three non-beetle insects (electronic supplementary material, table S4), we built and analysed orthogroups (gene families) using OrthoFinder [55]. We identified 17 135 gene families present in at least two species and a high percentage of genes grouped in families in beetles (85%–100%) and outgroup species (71%–85%) (electronic supplementary material, table S4).

To comprehensively examine gene family evolution in SPB and other *Dendroctonus* species, we analysed gains and losses in orthogroups along the phylogeny of the 14 beetles and three outgroup species using CAFE [57].

**Figure 4.**
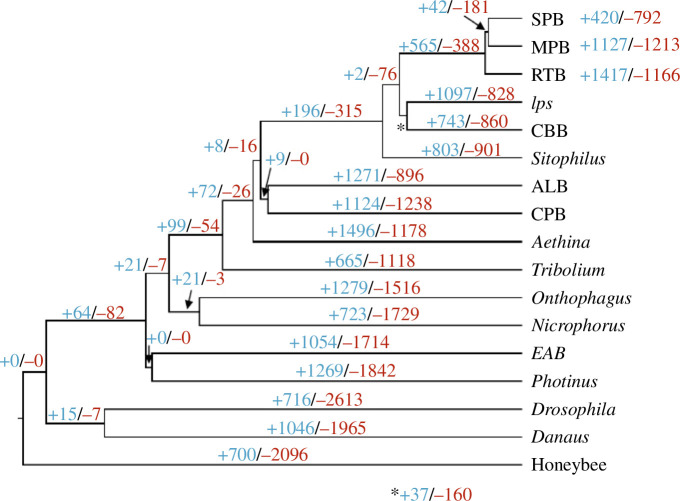
Gene family (orthogroups) changes along the phylogeny of 14 beetles and three outgroup insects inferred using CAFE. The number of gene families with gains and losses are shown in blue and red, respectively. SPB: southern pine beetle (*D. frontalis*). MPB: mountain pine beetle (*D. ponderosae*). RTB: red turpentine beetle (*D. valens*). Ips: Ips typographus. CBB: coffee berry borer (*Hypothenemus hampei*). Sitophilus: *Sitophilus oryzae*. ALB: Asian longhorned beetle (*Anoplophora glabripennis*). CPB: Colorado potato beetle*. Aethina: Aethina tumida. Tribolium: Tribolium castaneum. Onthophagus: Onthophagus taurus. Nicrophorus: Nicrophorus vespilloides.* EAB: emerald ash borer (*Agrilus planipennis*). *Photinus: Photinus pyralis. Drosophila: Drosophila melanogaster. Danaus: Danaus plexippus. Honeybee: Apis mellifera.*

In SPB and across the entire genus *Dendroctonus* and the subfamily Scolytinae, gene family contractions generally outnumbered expansions, with the exceptions of *Ips* and the ancestral *Dendroctonus* branch (figure 4). This is in agreement with the observed diminished gene content in *Dendroctonus* and increased gene count in *Ips* compared with other beetles. We next explored changes in gene family size in SPB, the ancestral branch of SPB and MPB, and the ancestral *Dendroctonus* lineage that could be relevant to the *D. frontalis* trait evolution and the prevalence of the tree-killing habit species in this genus of bark beetles.

The CAFE analysis revealed 792 families contracted in SPB; of these, 437 were apparently completely lost. Gene losses contribute significantly to species evolution and adaptation [63,64], but remain poorly investigated in insects. To better assess the magnitude and potential biological impact of gene loss in SPB, we carried out BLAST searches of MPB genes belonging to these families against the SPB genome assembly and confirmed the lack of any homology for eight orthogroups. As BLAST analyses were permissive in order to retrieve SPB regions with low homology to the MPB genes, these results could include false positives (see also §2). Nevertheless, these indicate that several genes might still be unreported or only partially annotated in the SPB gene set; alternatively, they might have incurred pseudogenization (gene loss via coding disabling mutations) in SPB. Genes missing in SPB included the serine/threonine-protein kinase *Tricornered*, a splicing factor, an amino acid transporter and a protein tyrosine phosphatase (electronic supplementary material, table S7).

Among the 420 orthogroups with gene gains in SPB, we examined the possible function of 293 families with more genes in SPB than other Scolytinae. Functional enrichment was determined using MPB and *D. melanogaster* members of these families (one gene per family) using the STRING database [56]. Genes encoding for membrane proteins and extracellular matrix proteins experience high rates of duplication in SPB, suggesting a key role of proteins at the cellular–environment interface in adaptation and specialization (electronic supplementary material, table S8). Results using MPB genes produced only one significant enrichment, which is expected given the limited functional annotation of most MPB genes, corresponding to the large ‘Cell periphery’ cellular component (electronic supplementary material, table S8). To further dissect the contribution of gene duplication to trait evolution in SPB, we analysed a subset of 85 orthogroups that contained two genes in SPB and one gene in other Scolytinae and in *D. melanogaster* (subset ‘2-to-1 SPB’). Three large partially overlapping networks, ‘Plasma membrane bounded cell projection organization’, ‘Cell morphogenesis involved in differentiation’ and ‘Animal organ development’, stood out as processes with significant gene family expansions in SPB (electronic supplementary material, table S8).

We next investigated contractions and expansions of gene families in the ancestral branch of SPB and MPB. A total of 141 gene families appeared extinct in both SPB and MPB compared with their sister *Dendroctonus* species RTB. BLAST searches revealed homologous hits in the SPB and MPB genomes for all but 11 of these 141 orthogroups. Several genes conserved across beetles and other insects were lost in SPB and MPB, including a locus required for the development of *D. melanogaster* ovarian follicles (*Kuduk*) and a gene regulating tube morphogenesis in the tracheal system (*Ccm3*) (electronic supplementary material, table S7). Notably, *Ccm3* genetically interact with *Tricornered* [65], one of the genes uniquely lost in SPB, implying significant changes in the control of tube morphogenesis in the *Dendroctonus* clade. Lineage-specific gene duplications in the SPB/MPB clade occurred only in 42 gene families but showed significant enrichment for ‘Cell junction’ and ‘Mitotic spindle’ processes (electronic supplementary material, table S8).

Along the *Dendroctonus* stem lineage, we retrieved 388 orthogroups with contractions, including 218 completely lost gene families. BLAST analyses using *Ips*, coffee berry borer (CBB) and *D. melanogaster* genes belonging to these families confirmed the loss of 17 orthogroups. The 23 *D. melanogaster* genes with no homologues in *Dendroctonus* contained highly conserved loci involved in survival to dietary restriction and oxidative stress (*Thor*), maintenance of the female germ line (*Stonewall* and *Brickwall*), transcription of mitochondrial proteins (*Spargel*), mitotic chromosome condensation (*prod* and *Mink*), and repair of UV-induced DNA damage (*phr*) (electronic supplementary material, table S7).

A total of 565 gene families showed gene gains in the *Dendroctonus* clade. We searched for functional enrichments in the 200 expanded families with the largest increase between the *Dendroctonus* and Scolytinae branches. Gene family expansions were associated with a variety of processes that might be involved in adaptation, including ‘Response to stimulus’, ‘Locomotion’ and ‘Compound eye development’ (electronic supplementary material, table S8).

### 3.6. Plant cell wall-degrading enzyme genes in southern pine beetle

PCWDEs are required to digest cellulose, pectin and other complex carbohydrates that constitute the plant cell wall, a major energy source for herbivorous insects [66]. A recent survey of Coleopteran transcriptomes and genomes showed correlated expansions in horizontally acquired PCWDEs with adaptive radiations and specialized herbivory [67]. PCWDE family expansions were particularly common among Phytophaga and Buprestoidea, the most taxonomically diverse and specialized lineages within Coleoptera. Using Pfam domain results we predicted a total of 651 PCWDE genes across the 17 species (electronic supplementary material, table S9). We found a very similar number of PCWDE genes in the wood-boring species MPB, Asian longhorned beetle (ALB) and emerald ash borer (EAB)compared with those previously described by McKenna *et al*. [67]. Our novel annotation of PCWDE genes in SPB and RTB confirmed a high number of these genes across *Dendroctonus*, albeit to a lesser extent than observed in MPB (electronic supplementary material, table S9).

### 3.7. Gene content reduction in *Dendroctonus* and gene misannotation in beetles

The three sequenced *Dendroctonus* species contain on average approximately 13 400 genes compared with a mean of approximately 17 000 genes in the other 11 beetle species. Furthermore, the gene count in each *Dendroctonus* species is lower than in any of the other beetle genomes, with the only exception of the burying beetle *Nicrophorus vespilloides*, which contains slightly fewer genes than *D. valens* (electronic supplementary material, table S4). Notably, 43%–79% more genes have been reported in the two other sequenced Scolytinae genomes, the spruce bark beetle and the coffee berry borer, than in *Dendroctonus*. We sought to disentangle the possible contribution of biological factors and gene annotation shortfalls to the diminished gene repertoire in *Dendroctonus* genomes.

First, we found a lower gene annotation completeness between *Dendroctonus* and other species in the suite of highly conserved Endopterygota genes assessed by BUSCO, confirming the gene family analysis results indicating loss of several conserved genes in *Dendroctonus* genomes (electronic supplementary material, table S10). *Dendroctonus* showed on average approximately 1% more missing conserved genes than other beetles, or approximately 160 loss genes after extrapolating to the average beetle gene count of approximately 17 000.

However, the BUSCO analysis is limited to a subset of genes that are unlikely to be representative of the overall gene complement of a species, particularly for gene families with high rates of turnover. Therefore, we expanded our analyses of gene family size to better determine how gene gains and losses shaped the gene content differences across beetles. The CAFE results showed that gene gain and loss rates were similar in *Dendroctonus* compared with other species (electronic supplementary material, figure S1). Moreover, the average size of orthogroups used in the CAFE analysis is nearly identical between *Dendroctonus* and other species (electronic supplementary material, table S11). Thus, we reasoned that gene content differences among these two groups of beetles must lie within the 9865 gene families present in beetles that were excluded from the CAFE analyses. Among these, the 3764 families occurring in *Dendroctonus* showed no difference in size between the two groups of beetles (electronic supplementary material, table S11). This suggests that gene content is higher in non-*Dendroctonus* species primarily due to orthogroups that do not occur in the *Dendroctonus* clade. Notably, nearly 83% of these orthogroups are present in less than three beetle genomes, indicating that they derive from the emergence of novel lineage-specific genes (electronic supplementary material, table S4). Additionally, only approximately 228 genes in *Dendroctonus* were not included in orthogroups, compared with approximately 1256 genes in non-*Dendroctonus* species, supporting the higher proportion of lineage-specific genes in the latter group (electronic supplementary material, table S11).

**Figure 5.**
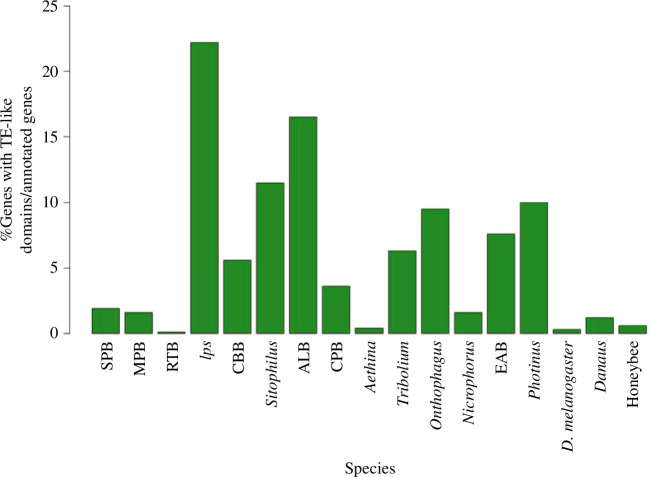
Proportion of functionally annotated genes encoding proteins with TE-derived domains. Name abbreviations are listed in electronic supplementary material, table S4.

We next sought to assess if the high number of lineage-specific genes in non-*Dendroctonus* beetles could be caused by assembly and annotation artefacts [68]. In particular, we investigated the potential role of transposable elements (TEs) as a source of gene annotation artefacts. Insect genomes harbour several genes that originated from the ‘domestication’ of TEs [69] but they typically form a small portion of the overall gene repertoire and should not account for major differences in gene counts between species. We developed a novel approach to rapidly screen the gene sets of all analysed species for the presence of an excess of TEs-derived genes. First, we identified proteins containing domains derived from TEs based on eggNOG-mapper annotation and searchers of TE-associated keywords. We observed a much higher number of genes containing TE-derived domains in the bark beetle species *Ips* and CBB compared with *Dendroctonus*, as well as in ALB, *Sitophilus*, *Onthophagus* and *Photinus* (figure 5; electronic supplementary material, table S12).

To verify if most of these genes represent misannotated TEs, we performed BLAST searches for each candidate TE-derived protein against their genome of origin and estimated their copy number using several combinations of sequence identity and distance between genomic hits (see §2). The same approach was used to estimate the copy number of the 12 largest gene families in our dataset that do not contain TE-derived domains. Putative genes with TE domains had more copies on average than non-TE genes in every species, with *Ips* having the highest copy number for the former (electronic supplementary material, table S12). These estimates might have been slightly inflated for non-TE genes due to multiple hits for the same gene within the distance range between hits. Additionally, we found a higher proportion of copies with stop codons between genes with TE domains compared with other genes in each species for most comparisons (electronic supplementary material, table S12). This is expected for misannotated TEs, as many copies of transposable elements contain disabled coding sequences.

We further assessed if TE misannotation could be responsible for the observed difference in the total gene number between *Dendroctonus* and non-*Dendroctonus* beetles. For each species, we extrapolated the expected total number of genes containing TE-like domains from the results of the eggNOG annotation (electronic supplementary material, table S12). Then, we subtracted these values from the total numbers of annotated genes and obtained adjusted gene counts. We found that even after adjusting for genes with TE-like domains, the genus *Dendroctonus* still averages approximately 2300 fewer genes than non-*Dendroctonus* beetles (electronic supplementary material, table S12).

Altogether, these results suggest that the diminished gene count in *Dendroctonus* is due to a combination of high levels of gene loss in this genus and a large apparent expansion of lineage-specific gene families in many non-*Dendroctonus* beetles, which is partly due to the misannotation of many TEs as genes. We argue that several potential biological explanations for these two phenomena exist. Gene losses might be higher in *Dendroctonus* due to the high levels of symbiotic interactions with bacteria and fungi reported in this genus [70–72]. It is possible that symbionts complement the metabolic repertoires of their beetle hosts, thus decreasing the selective pressure to maintain specific genes in the genome of *Dendroctonus* [73]. Comparative work with other Coleoptera and their symbionts could reveal more specific associations between symbiosis and gene count. Alternatively, the ecological specialization of tree-killing *Dendroctonus* species might have led to the loss of unnecessary genes. Genome sequencing and analyses of non-tree-killing *Dendroctonus* species will be necessary to disentangle the contribution of ecological and evolutionary factors to gene loss patterns across this genus.

Furthermore, *Dendroctonus* might experience a decreased propensity to form novel gene families due to multiple reasons. First, the *Dendroctonus* lineage is evolutionarily younger than other beetle taxa available for this study, a feature that can partly explain the lower number of genus-specific gene families. Second, new genes may arise at a lower rate in *Dendroctonus* than in most other Coleoptera. While gene duplication rates appear to be largely similar across beetles (electronic supplementary material, table S11), new gene evolution via other processes [74], including de novo gene birth and recruitment of transposable element genes, might occur infrequently in *Dendroctonus*. Evolutionary analyses of patterns of gene formation across the rapidly increasing number of beetle genomes hold the promise to discriminate between different scenarios potentially responsible for the decreased gene content in *Dendroctonus*.

## 4. Conclusions

Genome sequencing and analysis efforts are essential to identifying the genetic basis of pest behaviour in insects and to inform advanced pest management strategies. Using long-read genome sequencing and high-throughput transcriptomic data, we generated a chromosome-level assembly and high-quality gene annotation of the southern pine beetle *Dendroctonus frontalis*, a major conifer pest. We confirmed the extensive synteny conservation across beetles and identified the putative X chromosome in SPB. Gene family analyses of 14 beetle species revealed several losses of conserved genes and lineage-specific gene gains in SPB and other *Dendroctonus* species. Overall, the *Dendroctonus* clade experienced numerous gene losses and a reduced rate of formation of novel gene families, which seem to account for the diminished gene complement in this genus. However, we found strong evidence of widespread misannotation of TEs in the gene complement of many non-*Dendroctonus* beetles, which could adversely affect analyses of gene and genome evolution in Coleoptera. The non-*Dendroctonus* species analysed in our study showed a variety of ecological and life history features and include some taxa with wood-boring habits, such as *Ips typographus*, the Asian longhorned beetle and the emerald ash borer. This suggests that the gene repertoire reduction in *Dendroctonus* might be uniquely associated with the evolutionary history of this genus. An elevated functional contribution of genes from symbionts and the lower propensity to form new genes might further contribute to the diminished gene complement of tree-killing *Dendroctonus* bark beetles.

## Data Availability

The *Dendroctonus frontalis* genome assembly sequence is available through the NCBI BioSample ID PRJNA1100959. Raw transcriptome sequencing reads are available through the SRA ID PRJNA1102401. Datasets and gene family analysis results are available through the following Figshare repository [75]. Supplementary material is available online [76].
